# Estimation of Full-Length TprK Diversity in Treponema pallidum subsp. *pallidum*

**DOI:** 10.1128/mBio.02726-20

**Published:** 2020-10-27

**Authors:** Amin Addetia, Michelle J. Lin, Quynh Phung, Hong Xie, Meei-Li Huang, Giulia Ciccarese, Ivano Dal Conte, Marco Cusini, Francesco Drago, Lorenzo Giacani, Alexander L. Greninger

**Affiliations:** a Department of Laboratory Medicine, University of Washington, Seattle, Washington, USA; b Health Sciences Department, Section of Dermatology, San Martino University Hospital, Genoa, Italy; c STI Clinic, Amedeo di Savoia Hospital, University of Turin, Turin, Italy; d Fondazione IRCCS Ca' Granda, Ospedale Maggiore Policlinico, Milan, Italy; e Department of Medicine, University of Washington, Seattle, Washington, USA; McGovern Medical School

**Keywords:** PacBio, *T. pallidum*, gene conversion, immune evasion, syphilis, *tprK*, treponema

## Abstract

Immune evasion and disease progression of Treponema pallidum subsp. *pallidum* are associated with sequence diversity in the hypervariable outer membrane protein TprK. Previous attempts to study variation within TprK have sequenced at depths insufficient to fully appreciate the hypervariable nature of the protein, failed to establish linkage between the protein’s seven variable regions, or were conducted on isolates passed through rabbits. As a consequence, a complete profile of *tprK* during infection in the human host is still lacking. Furthermore, prior studies examining how T. pallidum subsp. *pallidum* uses its repertoire of genomic donor sites to generate diversity within the variable regions of the *tprK* have yielded a partial understanding of this process due to the limited number of *tprK* alleles examined. In this study, we used short- and long-read deep sequencing to directly characterize full-length *tprK* alleles from T. pallidum subsp. *pallidum* collected from early lesions of patients attending two sexually transmitted infection clinics in Italy. We demonstrate that strains collected from cases of secondary syphilis contain significantly more unique variable region sequences and full-length TprK sequences than those from cases of primary syphilis. Our data, combined with recent data available on Chinese T. pallidum subsp. *pallidum* specimens, show the near-complete absence of overlap in TprK sequences among the 41 specimens profiled to date. We further estimate that the potential antigenic variability carried by TprK rivals that of current estimates of the human adaptive immune system. These data underscore the immunoevasive ability of TprK that allows T. pallidum subsp. *pallidum* to establish lifelong infection.

## Abstract (precis)

Syphilis continues to be a significant public health issue in both low- and high-income countries, including the United States where the rate of syphilis infection has increased over the past 5 years. Treponema pallidum subsp. *pallidum*, the causative agent of syphilis, carries the outer membrane protein TprK that undergoes segmental gene conversion to constantly create new sequences. We performed full-length deep sequencing of TprK to examine TprK diversity in clinical T. pallidum subsp. *pallidum* strains. We then combined our results with data from all samples for which TprK deep sequencing results were available. We found almost no overlap in TprK sequences between different patients. Moreover, our data allowed us to estimate the total number of TprK variants that T. pallidum subsp. *pallidum* can potentially generate. Our results support how the T. pallidum subsp. *pallidum* TprK antigenic variation system is an equal adversary of the human immune system leading to pathogen persistence in the host.

## INTRODUCTION

Syphilis, caused by the spirochete Treponema pallidum subsp. *pallidum*, is a significant global health problem. Although most syphilis cases occur in low-income countries, where the disease is endemic, rates of syphilis infection have been steadily increasing for the last 2 decades in high-income nations, particularly in men who have sex with men (MSM) and HIV-infected individuals (1, 2). Syphilis is a chronic sexually transmitted infection marked by distinct early and late stages (3). These stages are generally distinguished by unique clinical manifestations, with symptoms associated with the late stage developing up to several decades after initial infection and following a long period of latency (4).

The mechanisms that allow T. pallidum subsp. *pallidum* to persist for the lifetime of an infected individual are not fully understood. During natural and experimental syphilis infection, a robust host immune response is developed against T. pallidum subsp. *pallidum* (5–7). This suggests immune evasion strategies developed by T. pallidum subsp. *pallidum* are a key aspect of its pathogenesis (8).

The ability of T. pallidum subsp. *pallidum* to evade the host immune response is attributed to the organism’s scarcity of surface-exposed outer membrane proteins (OMPs), prolonged generation time (∼33 h), and ability to stochastically and rapidly switch on and off the expression of genes encoding putative OMPs through phase variation (9). Chief among the immune evasion strategies evolved by T. pallidum subsp. *pallidum* is its ability to generate diversity within the OMP TprK (10–12). TprK harbors seven discrete variable (V) regions, namely, V1 to V7. In the putative TprK beta-barrel structure, each variable region is predicted to form a loop exposed at the host-pathogen interface (13). The generation of variants in these V regions occurs through nonreciprocal segmental gene conversion, a process in which sections from donor sites flanking the *tprD* gene (*tp0131*) are stitched together to create new sequences (14, 15). Forty-seven putative donor sites have been identified thus far (14); however, the total number of unique TprK sequences that can be generated in a T. pallidum subsp. *pallidum* strain has yet to be determined.

Gene conversion results in significant intra- and interstrain diversity of the TprK protein (14–19). In rabbit models, diversity in TprK actively accumulates over the course of an infection and appears to be a result of selection of the host’s immune response (18, 20). At least five of the variable regions, namely, V2 and V4 to V7, elicit an antibody response in rabbit models (21). These antibodies are specific for a single variable sequence, which further supports that generation of new V region sequences allows T. pallidum subsp. *pallidum* to evade the host response. Furthermore, an increased diversity of TprK is directly correlated with more advanced stages of syphilis (17, 22). In both rabbit models and humans, T. pallidum subsp. *pallidum* strains isolated from cases of secondary/disseminated syphilis contained more TprK diversity than those isolated from cases of primary syphilis (17, 22).

Previous studies to evaluate TprK variability within T. pallidum subsp. *pallidum* strains have sequenced a limited number of TprK clones, failed to resolve linkage between variable regions, or been conducted on strains passed through rabbits (16–19, 23). As a result, no studies to date have adequately profiled TprK within T. pallidum subsp. *pallidum* during infection in the human host. Furthermore, an understanding of how different donor sites contribute to variable region sequences has been hindered by the analysis of a limited number of *tprK* clones (14). In this study, we used short- and long-read deep sequencing to directly characterize TprK in T. pallidum subsp. *pallidum* collected from early genital or anal lesions of 13 individuals attending two sexually transmitted infection clinics in Milan and Turin in Italy (24). We then combined our data with recent short-read *tprK* sequencing data from 28 T. pallidum subsp. *pallidum* specimens collected in China to illustrate the near-complete lack of overlap in TprK sequences among all 41 clinical specimens directly and deeply profiled to date. Moreover, our data help to redefine the TprK variable regions and provide an estimate of the number of TprK variants that T. pallidum subsp. *pallidum* can potentially generate with its repertoire of donor cassettes. Overall, our data reiterate the pivotal importance of the TprK antigenic variation system to allow T. pallidum subsp. *pallidum* persistence in the host during infection.

## RESULTS

### Italian patient metadata.

We selected 13 T. pallidum subsp. *pallidum* specimens collected from syphilis patients, comprising 7 primary and 6 secondary syphilis cases, in Milan and Turin in Italy (Table 1; see Table S1 in the supplemental material). All patients reported to be MSM, and the median age of individuals was 39 years (range, 20 to 57 years). Eight of the individuals sampled were HIV positive, and for nine of the patients, this was the first syphilis diagnosis. Seven of the specimens were collected from genital lesions, while the remaining six were collected from anal lesions.

**TABLE 1:** Summary statistics of patient metadata for strains sequenced in this study

Parameter	% (*n* = 13)
Location	
Milan	30.77
Turin	69.23
Stage	
Primary	53.85
Secondary	46.15
Strain type	
14d/g	61.54
13d/g	23.08
13d/d	7.69
6d/f	7.69
Gender	
Male	100
Sexual orientation	
MSM	100
Age	
Median (Min–Max)	39 (20–57)
HIV status	
Positive	61.54
Negative	38.46
Lesion location	
Genital	53.85
Anal	46.15
Genotypic antibiotic resistance	
Tetracycline resistance	0
Macrolide resistance	100
Infection status	
First time infected	69.23
Previous infection	30.77

10.1128/mBio.02726-20.5 TABLE S1 Individual metadata for strains sequenced in this study. Download Table S1, XLSX file, 0.01 MB. Copyright © 2020 Addetia et al. 2020 Addetia et al. This content is distributed under the terms of the Creative Commons Attribution 4.0 International license.

### TprK diversity in T. pallidum subsp. *pallidum* specimens directly sampled from individuals.

Through short-read sequencing, we identified a median of 54 (range, 30 to 135) unique sequences from all 7 V regions from our 13 T. pallidum subsp. *pallidum* strains. Across the 13 strains, V4 contained the fewest unique sequences (median, 3; range, 1 to 8), while V1, as determined by the Shannon diversity index, was the least diverse variable region (median, 0.077; range, 0.012 to 0.869). V6 contained the greatest number of unique variants (median, 15; range, 4 to 54) and was also the most diverse variable region (median, 1.044; range, 0.241 to 2.617) (see Table S2 in the supplemental material).

10.1128/mBio.02726-20.6 TABLE S2 Number of variable region sequences and diversity measures for the 7 variable regions of TprK for the 13 strains profiled in this study. Download Table S2, XLSX file, 0.01 MB. Copyright © 2020 Addetia et al. 2020 Addetia et al. This content is distributed under the terms of the Creative Commons Attribution 4.0 International license.

**FIG 1:**
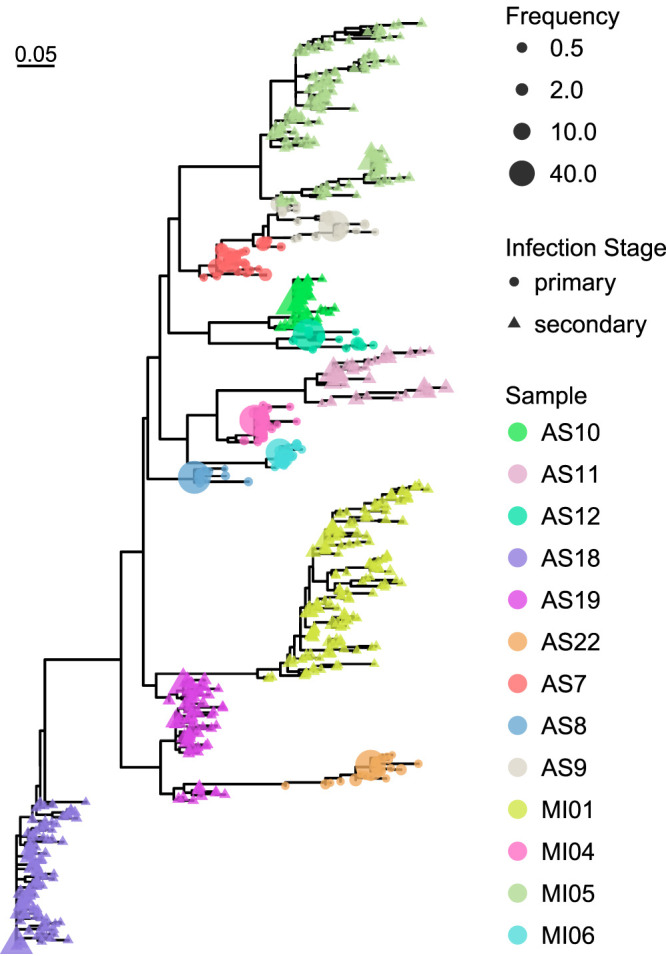
Full-length TprK phylogeny of all high-confidence protein sequences from 13 patients from Italy. Only intact, high-confidence, full-length TprK sequences derived from PacBio sequencing were used to generate the phylogenetic tree. Each individual is labeled by a different color, the stage of syphilis infection is represented by shape, and the proportion of sequences is shown by node size. None of the 629 full-length TprK sequences were shared between the 13 T. pallidum subsp. *pallidum* specimens sequenced in this study.

Using our long-read data, we recovered a total of 629 full-length TprKs across the 13 samples, ranging from 11 to 133 different full-length TprKs within each sample (see Data Set S1 in the supplemental material). The most prevalent TprK in each sample was generally located near the root of the TprK phylogenetic tree for that particular sample (Fig. 1). Notably, none of the 629 full-length TprKs were shared between the 13 profiled specimens.

10.1128/mBio.02726-20.2 Data Set S1 XLS file containing Illumina-verified, phased TprK variable regions obtained from long-read sequencing of the TprK amplicons. Download Data Set S1, XLSX file, 0.04 MB. Copyright © 2020 Addetia et al. 2020 Addetia et al. This content is distributed under the terms of the Creative Commons Attribution 4.0 International license.

We next examined the diversity of TprK in the context of different clinical characteristics. T. pallidum subsp. *pallidum* strains collected from cases of secondary syphilis contained significantly more unique variable region sequences (*P* = 0.002) and significantly more full-length TprK sequences (*P* = 0.003) and were significantly more diverse (*P* = 0.005) than those strains collected from cases of primary syphilis (Fig. 1; Table 2). The number of unique variable region sequences or full-length sequences did not significantly differ (*P* = 0.181; *P* = 0.224) between strains collected from anal or genital lesions. However, specimens collected from anal lesions exhibited significantly more diversity (*P* = 0.035) across the seven V regions. This difference in the variable region diversity between specimens collected from anal and genital lesions is likely due to the greater percentage of secondary syphilis cases associated with anal specimens (67% of anal lesions from secondary syphilis cases versus 29% of genital lesions from secondary syphilis cases). Specimens collected from HIV-positive individuals exhibited significantly more variable region sequences (*P* = 0.045). In contrast, the number of full-length TprK sequences and the total diversity across the seven variable regions did not significantly differ between HIV-positive and HIV-negative individuals (*P* = 0.272; *P* = 0.171). The difference in the number variable region sequences between specimens collected from HIV-positive and HIV-negative individuals could be also attributed to the greater percentage of secondary syphilis cases in HIV-positive individuals (83% of HIV-positive individuals with secondary syphilis versus 43% of HIV-negative individuals with secondary syphilis). No significant differences were observed in the number of unique variants, number of full-length sequences, or diversity when stratified by history of prior T. pallidum subsp. *pallidum* infection (*P* = 0.604; *P* = 0.938; *P* = 0.604).

**TABLE 2:** Comparison of the sequences and diversity across 7 variable regions of TprK

Parameter	No. of strains	Total no. of variable region sequences		Total no. of full-length TprK sequences		Total diversity	
		Median (range)	*P* value	Median (range)	*P* value	Median (range)	*P* value
Stage							
Primary	7	39 (30–59)	0.002	22 (11–26)	0.003	2.01 (0.78–3.93)	0.005
Secondary	6	90 (54–135)		80 (35–133)		6.21 (3.39–9.08)	
HIV status							
Positive	8	73.5 (39–135)	0.045	48 (14–133)	0.272	3.92 (2.01–9.08)	0.171
Negative	5	36 (30–59)		26 (11–37)		1.91 (0.78–6.48)	
Lesion location							
Genital	7	43 (30–82)	0.138	26 (11–99)	0.224	2.01 (0.78–4.46)	0.022
Anal	6	76 (39–135)		49 (14–133)		6.21 (3.29–9.08)	
Infection status							
First time infected	9	54 (30–116)	0.604	26 (11–133)	0.38	3.39 (0.78–9.08)	0.604
Previous infection	4	62.5 (39–135)		43 (17–99)		3.92 (2.01–8.45)	
Passaged in rabbits							
Yes	2^*a*^	199.5 (161–238)	0.019	243 (146–340)	0.034	12.04 (8.76–15.33)	0.038
No	13	54 (30–135)		26 (11–133)		3.39 (0.78–9.08)	

a Isolates were previously profiled by Addetia et al. (23).

In a previous investigation, we profiled TprK in two T. pallidum subsp. *pallidum* isolates (UW-148B and UW-148B2) collected from a single patient and amplified by two passages of strains in New Zealand White rabbits (23). To assess the impact of the additional passage through rabbits on TprK, we compared the number of unique variants and diversity across the 7 V regions identified from the 13 Italian T. pallidum subsp. *pallidum* strains and our 2 previously profiled strains. Using the dual Illumina library preparation strategy described here, these two strains we previously passed through rabbits contained a significantly greater number of variable region sequences (median, 199.5 versus 54; *P* = 0.019), significantly more full-length TprK sequences (median, 243 versus 26; *P* = 0.034), and significantly greater diversity across the seven variable regions (median, 12.04 versus 3.39; *P* = 0.038) than those of the 13 Italian T. pallidum subsp. *pallidum* strains.

### Comparison of TprK diversity between Italian and Chinese specimens.

**FIG 2:**
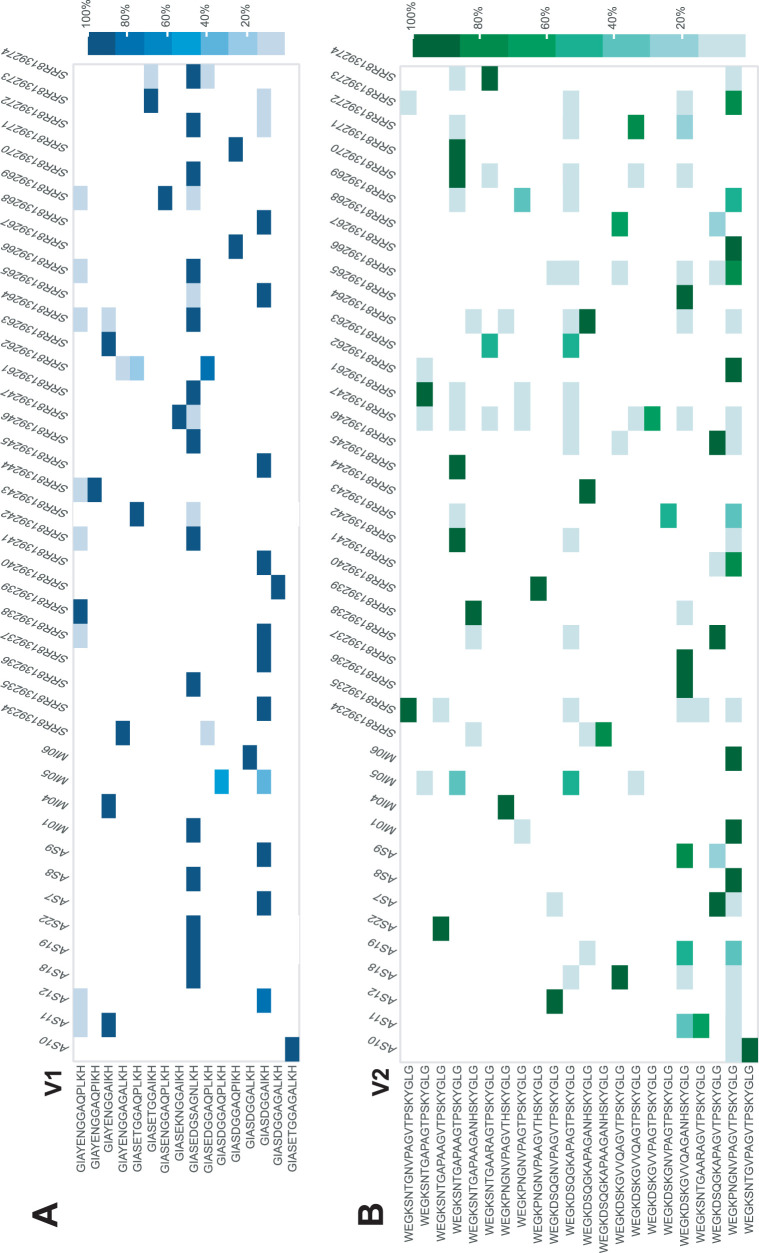

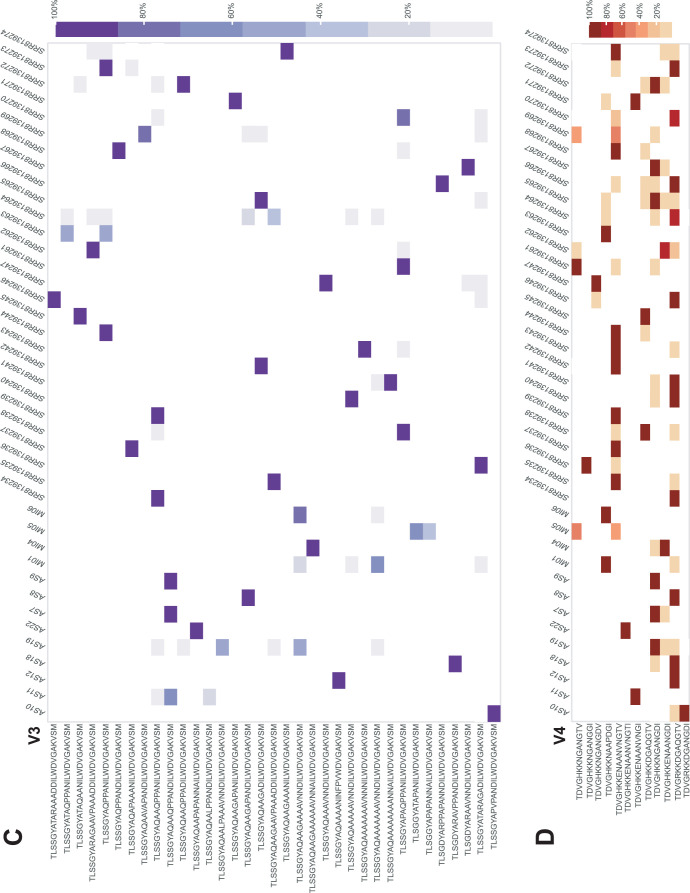

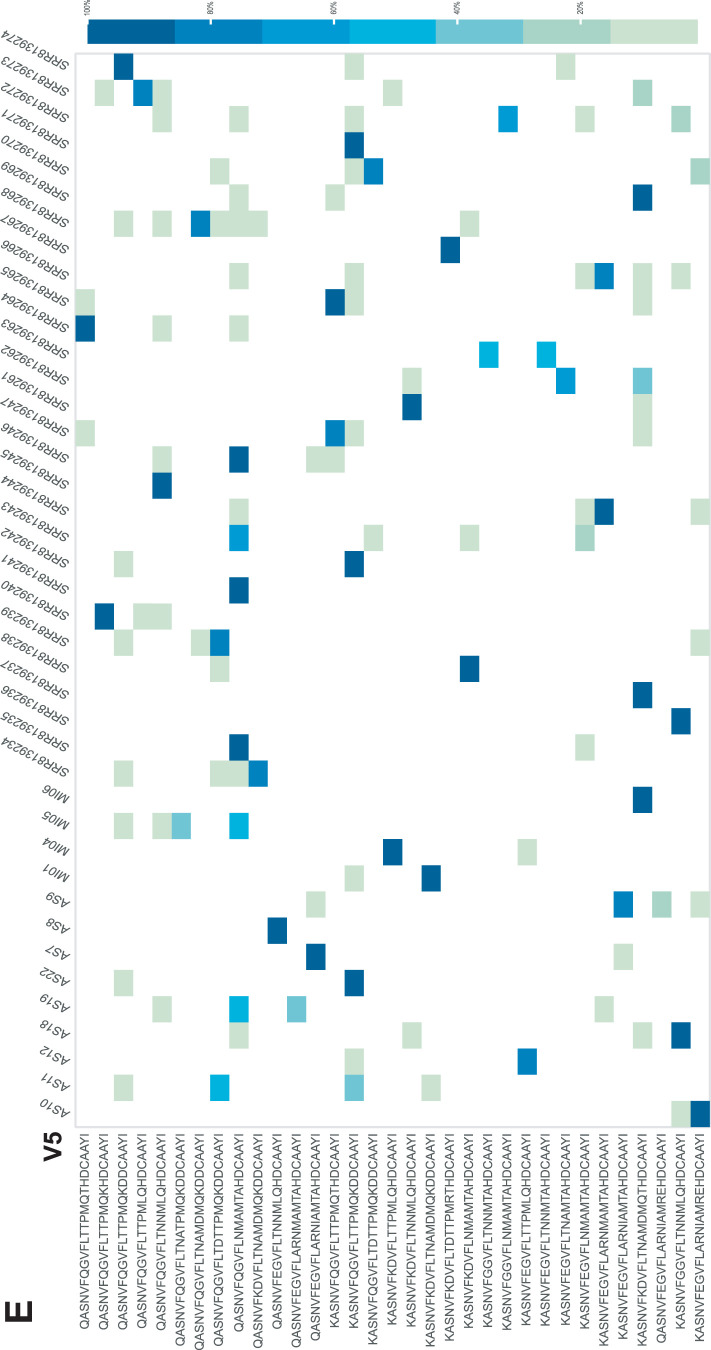

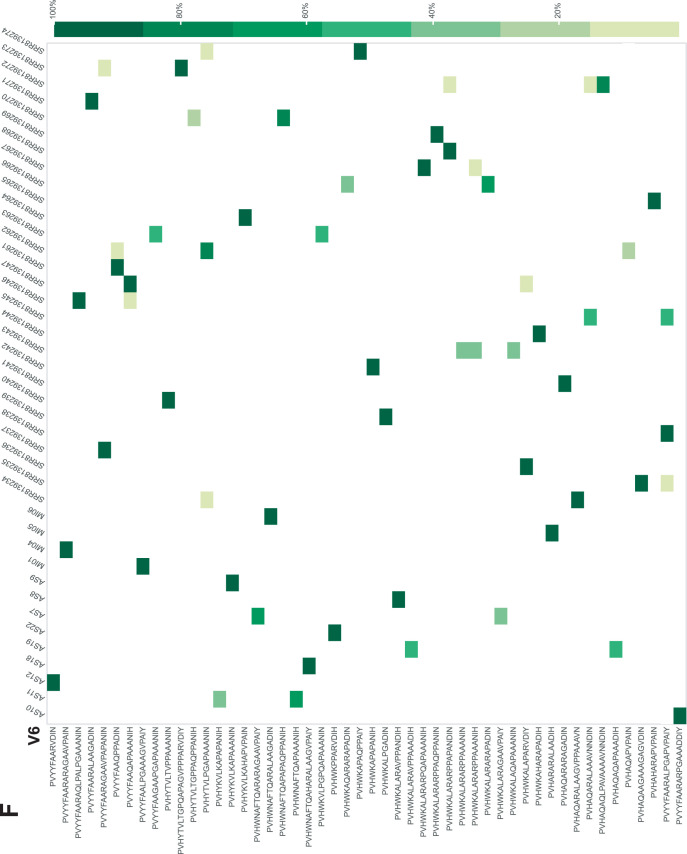

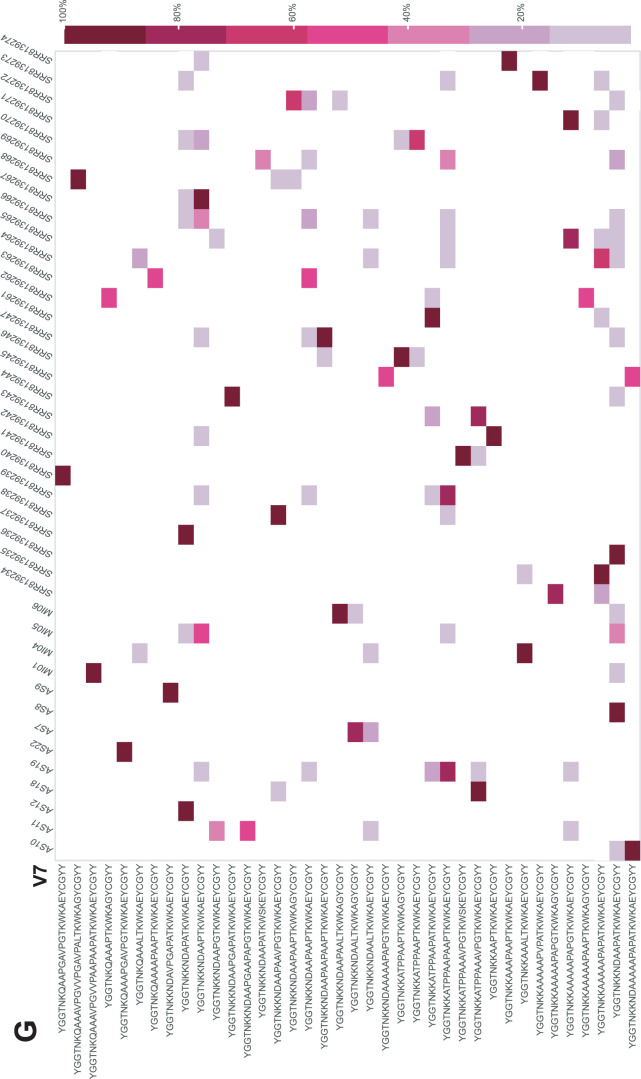
TprK variable region sequence heatmap. Heatmap display of all deep-sequenced *tprK* from clinical specimens to date, comprising 13 individuals from Italy sequenced here and 28 Chinese individuals from prior work. For print display, only those variable region sequences present at ≥20% frequency within a sample are depicted. Any variable frequencies less than 2% in other samples are not shown. The proportion of sequences is illustrated by color for each heatmap for V1 (A), V2 (B), V3 (C), V4 (D), V5 (E), V6 (F), and V7 (G). A heatmap filtered at a frequency of 1% is provided as an interactive html in Data Set S2.

We next examined whether the TprK V region sequences present in our 13 Italian individuals shared any overlap with TprK sequences derived from short-read sequencing of 28 T. pallidum subsp. *pallidum* specimens collected from cases of primary or secondary syphilis recently reported from China (16, 17). Given the extraordinary diversity present in *tprK*, for print display, we filtered out any variable sequences constituting <20% of the species present in a given sample (Fig. 2). More complex data filtered with a minimum frequency of 1% are displayed in an interactive figure in Data Set S2 in the supplemental material.

10.1128/mBio.02726-20.3 Data Set S2 Interactive HTML heatmap of TprK variable region frequencies across 13 Italian individuals and 28 Chinese individuals. The file contains any variable region sequence present in at least one strain at a frequency greater than 1%. Download Data Set S2, HTML file, 0.1 MB. Copyright © 2020 Addetia et al. 2020 Addetia et al. This content is distributed under the terms of the Creative Commons Attribution 4.0 International license.

The heatmap shows the impressive diversity present across the TprK variable regions. V1 and V4 were the most conserved (Fig. 2). The same two V1 sequences comprised the highest frequency species present in 8/13 (61.5%) Italian specimens and 16/28 (57.1%) Chinese specimens. Only 12 dominant V4 sequences were present across the 41 specimens. However, the most common V4 sequence present in the Chinese samples was represented only once in the Italian cohort at a frequency of >1%, and even then it was not the major species present.

The V3, V5, V6, and V7 regions demonstrated almost no overlap among the 41 specimens (Fig. 2). Only 8 of 32 dominant V7 sequences were shared between any Italian and any Chinese specimen. Notably, the highest frequency V6 sequence differed between all 41 specimens.

### Redefining conserved and variable regions in *tprK*.

The sequences we mined from variable regions were initially based off prior definitions of the conserved and variable portions of *tprK*, which themselves were based off comparatively few *tprK* sequences (14). While identifying donor sites, we noticed systematic biases in variable region sequence lengths mined from sequencing reads and the total blastn HSP length (see Fig. S1A and B in the supplemental material; reflected in Fig. 2). For instance, 100% of the V3 region sequences ended with the same 23-bp sequence (5′-TGTCGGGGCTAAGGTGAGTATGA-3′). Similarly, 100% of V5 region sequences ended with the same 13-bp sequence (5′-TGCTGCCTATATT-3′), and no V5 sequence had less than a 13-bp difference in sequence and blast hit length. For V2, 99.5% of sequences ended with the same 14-bp sequence (5′-AGTATGGATTGGGG-3′), and the alternative sequences could be explained by low-frequency Illumina sequencing error associated with G-quadruplexes (25). Removal of these sequences improved the ability to align *tprD* donor sites across the length of *tprK* variable region sequences, leaving a four-nucleotide common sequence (5′-TAGG-3′) in V4 region sequences that we left based on its short nature (Fig. S1C and D).

10.1128/mBio.02726-20.1 FIG S1 Blast sequence length alignment versus variable region sequence length plots before and after variable region sequence filtering. (A) Scatterplot of total sequence length alignment versus variable region sequence length after filtering of V2, V3, and V5 sequences of likely conserved region sequences. (B) Corresponding histogram of differences in total sequence length and alignment length after filtering. (C) Scatterplot of total sequence length alignment versus variable region sequence length based on prior definitions of variable region sequences. (D) Corresponding histogram of differences in total sequence and alignment length without filtering. Counts are absolute sequencing read counts across all 41 samples. Download FIG S1, PDF file, 0.03 MB. Copyright © 2020 Addetia et al. 2020 Addetia et al. This content is distributed under the terms of the Creative Commons Attribution 4.0 International license.

### Contribution of donor sites to variable regions.

**FIG 3:**
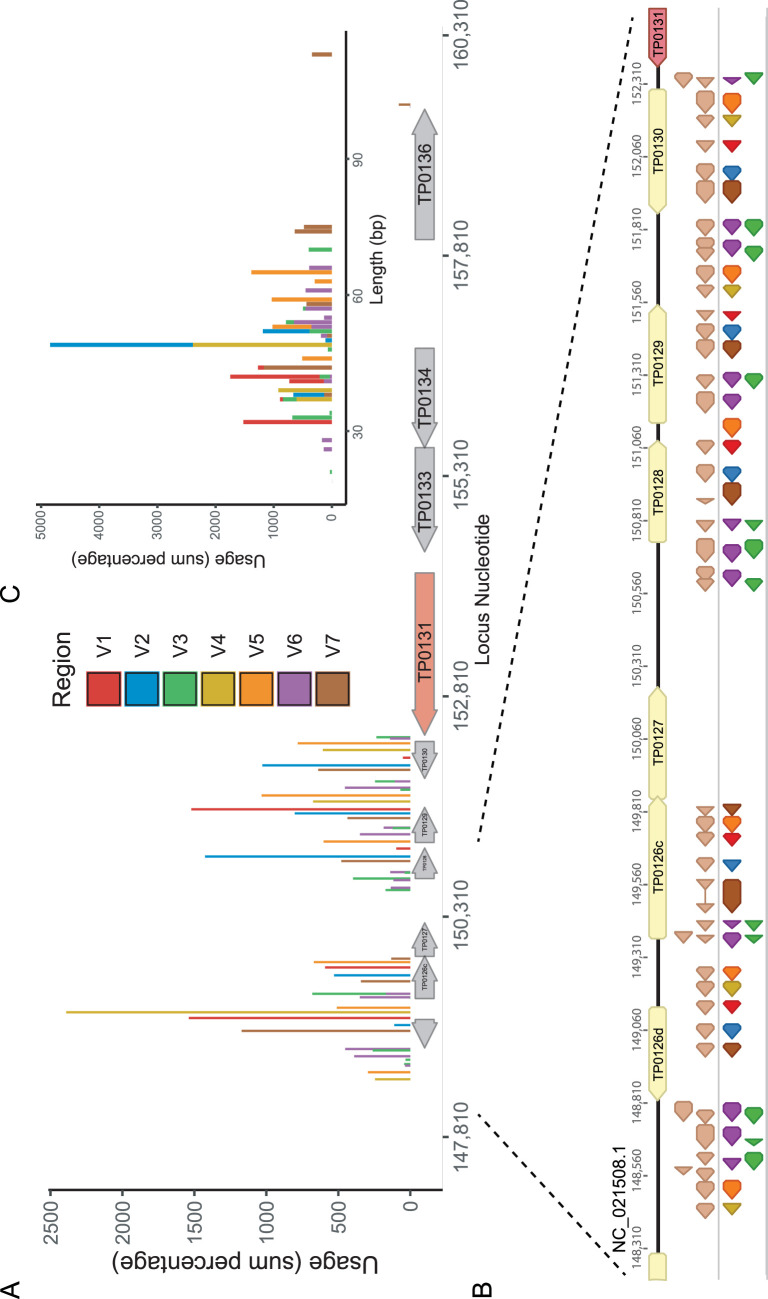
Map of *tprK* donor sites flanking the *tprD* locus. Variable region sequences were blastn aligned against a 12.5-kb locus that contained putative *tprK* donor sites based on manual review. (A) The usage of all 53 donor sites across the *tprD locus* by variable region is depicted based on the sum of within-sample percentages across all 41 samples. The nucleotide numbering of the *tprD* locus is based on the reference strain SS14 (NC_021508.1). (B) Zoomed-in depiction of the locus immediately downstream of *tprD* containing *tprK* donor sites. Donor sites are in the same orientation as the *tprD* locus. The light-brown sites include 45 of the 47 donor sites reported previously by Centurion et al. (14). The bottom donor sites include 51 of the 53 donor sites found in this study and are colored based on their associated variable region. (C) Length in nucleotides of the 53 donor sites identified from the analysis of *tprK* deep sequencing data from 41 clinical T. pallidum subsp. *pallidum* strains. The usage, represented as a sum percentage, as well as the variable region of each donor site is also depicted. The GFF file of the donor site locus is included as Data Set S3.

We next examined how each variable region sequence was generated from different donor sites using data from all 41 samples. We found a total of 53 donor sites, corresponding to 5 for V1, 5 for V2, 12 for V3, 4 for V4, 6 for V5, 14 for V6, and 7 for V7 (Fig. 3A). Forty-seven sites were previously reported by Centurion et al. (14). There was considerable overlap between the two sets, suggesting a finite limit to the number of donor sites for *tprK.* Of note, we did not identify a partial or full match to the previously described V2 donor site V2-DS45 within the variable region sequences of the 41 strains examined. The vast majority of the donor sites found in this analysis, namely, 51/53, were clustered downstream of *tprD*, while the remaining 2 donor sites were located upstream of *tprD*. Notably, all 51 of the donor sites located downstream of *tprD* were in the same orientation as *tprD* and had the highest utilization, while the 2 sites upstream of *tprD* faced in the opposite orientation. Donor sites for specific variable regions were collocated together, such as V1/V4/V5, V2/V7, and V3/V6. V3/V6 donor sites were almost uniformly derived from overlapping sequences (Fig. 3B). Donor sites for V1 and V4 were the shortest, measuring an average of 39.2 and 41.0 nucleotides, while V5 and V7 donor sites were the longest at 58.5 and 64.7 nucleotides (Fig. 3C).

10.1128/mBio.02726-20.4 Data Set S3 GFF file of *tprD* locus used in this study with previous donor sites and newly annotated donor sites. Download Data Set S3, TXT file, 0.003 MB. Copyright © 2020 Addetia et al. 2020 Addetia et al. This content is distributed under the terms of the Creative Commons Attribution 4.0 International license.

As we used the *tprD* locus from the T. pallidum subsp. *pallidum* strain SS14 (GenBank accession no. NC_021508.1) as our reference sequence for identifying donor sites, we performed whole-genome sequencing of four of the Italian specimens AS9, AS10, AS11, and AS12 to understand if sequence variation within the *tprD* locus can impact TprK diversity. The 12.5-kb *tprD* loci in one of the specimens, AS9, was identical to that of SS14. AS10 contained an insertion in a homopolymeric tract (156891insG), while AS12 contained a deletion in a homopolymeric tract (154198delC). The final specimen AS11 contained 1 single nucleotide variant (SNV) in the *tprD* locus, 153065T>A. These indels and SNVs were located outside the 53 donor sites we identified. Two donor sites (V1-DS15 and V2-DS21) previously identified by Centurion et al. had SNVs compared with our reference sequence but exactly matched their previously deposited *tprD* locus (GenBank accession no. AY587909.1) (14), indicating that chromosomal mutations in donor sites can affect *tprK* variable region sequences.

### Estimate of total potential diversity of *tprK*.

**FIG 4:**
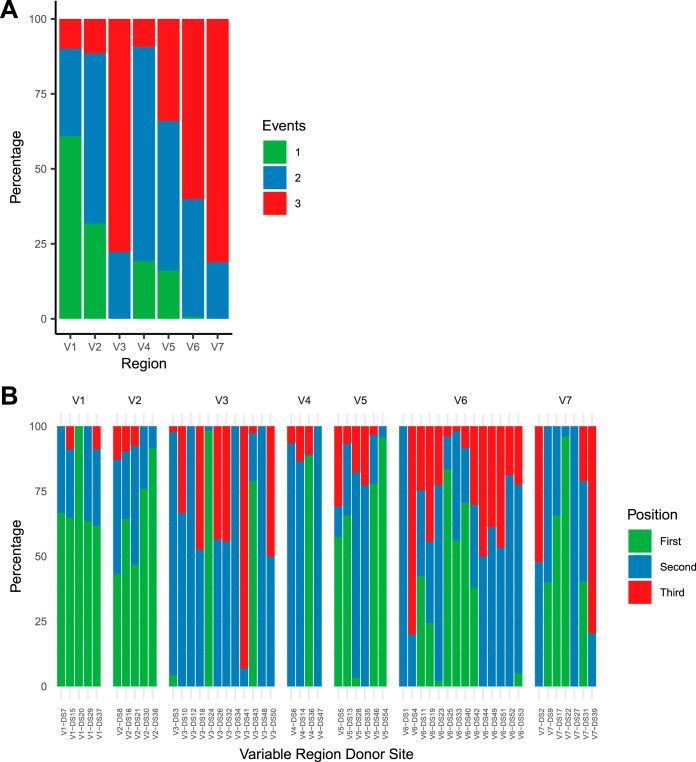
Donor site segments and position by V region. (A) The number of donor site contribution segments in each high-confidence variable region sequence was determined in blastn output across the 41 samples. Usage was determined by the sum percentage of variable region sequences by segment. For instance, V1 has the greatest number of variable region sequences where only one donor site segment is used in a given V region sequence, consistent with its overall lack of diversity. (B) The position of donor site contributions within a variable region sequence was also determined for each donor site (i.e., “first” means the donor site was found to align to the 5′-most segment of the variable region sequence, “second” means the donor site was found to align to the central segment of the variable region sequence, and “third” to the 3′-most segment of the sequence). Within-sample percentages were summed for each variable region in order to adjust for differences in read coverage at each locus between samples. These summed percentages were then adjusted by the total summed percentage to add up to 100% for each variable region.

Using this new inventory of *tprK* donor sites flanking the *tprD* gene, we next estimated the total coding diversity of TprK. Assuming a simple model in which only 1 donor site contributes to each variable region sequence, the 53 *tprD* donor sites across 7 variable regions could combine to create a total of 705,600 different full-length TprK sequences. However, multiple donor sites can contribute material to the same *tprK* variable region to create a mosaic variable region. Our manual review of donor site contributions to variable regions suggested that donor sites were limited to three separate contributions to create mosaic variable regions, so we set a limit of three for the number of high-scoring pairs in our blastn analysis of donor sites against each variable region sequence. The majority of V1 region sequences had only one donor site contribute to the sequence while no V3 or V7 sequences were generated by only one donor site (Fig. 4A). However, all variable regions had the potential for three donor site contributions. Adding up all potential combinations of one-, two-, and three-segment gene conversions that generate different sequences (assuming no single-segment V3 and V7 sequences) and assuming independence between variable regions lead to a potential diversity of TprK of 1.13 × 10^18^ full-length protein sequences if donor sites are reused or 3.93 × 10^16^ protein sequences without reuse (see Table S3 in the supplemental material).

10.1128/mBio.02726-20.7 TABLE S3 Combinations of TprK sequences. Download Table S3, XLSX file, 0.01 MB. Copyright © 2020 Addetia et al. 2020 Addetia et al. This content is distributed under the terms of the Creative Commons Attribution 4.0 International license.

We next examined whether certain donor sites were not represented in specific sections of a given variable region. Consistent with the segment usage data in Fig. 4A and B, we found biases in donor site contribution in every variable region. For instance, every V4 sequence starts with contributions from the same donor site and only two of five total V1 donor sites contribute to the third segment in V1. In addition, V3 and V6 regions make use of almost all of their donor sites in both the second and third segments but make use of considerably fewer potential donor sites in the first segment. Taking into account the differential use of donor sites by variable region segment reduced the potential total diversity to 1.23 × 10^15^ full-length TprK sequences with reuse of donor sites or 5.48 × 10^13^ sequences without reuse. Across 1,543 individual high-confidence variable region sequences, we found 145 variable region sequences that used the same donor site more than once in the same variable region sequence, indicating that some donor site reuse is allowed in the generation of *tprK* variable regions.

## DISCUSSION

Here, we combine deep, full-length profiling of TprK from T. pallidum subsp. *pallidum*-positive patient specimens with data mining of additional TprK short-read sequencing from 28 Chinese patients to explore the diversity of the outer membrane protein TprK. We find minimal overlap of specific variable regions within and between each patient cohort. None of the 629 high-quality, full-length TprK sequences were shared among any samples in the 13 patients on which we performed long-read sequencing. Consistent with previous reports, we found greater TprK diversity to be associated with secondary syphilis compared with primary syphilis (17, 22). We then used this data set of TprK diversity to find additional donor sites and to piece together the grammar of variable region generation.

Based on the lexicon of *tprK* donor sites measured using deep sequencing across 41 samples, we estimate a potential full-length TprK diversity approaching 10^13^ to 10^18^ proteins, assuming independence across donor sites. These estimates may be overestimates if our assumption of independence between variable region sequences is incorrect. These estimates may also underestimate the total diversity potential of TprK due to various lengths of donor site contributions to variable regions. Last, we cannot exclude the possibility that some of the TprK variants may reduce treponemal fitness to the point of being practically considered lethal variants. This possibility would further limit the repertoire of full-length TprKs that could be generated. Regardless, this antigenic variation is similar to if not greater than measures of the human adaptive immune system (26–28).

Our data also provide insights into differences in measured diversity among different variable regions. The limited diversity in V1 is associated with a greater use of single-segment gene conversions to generate the variable region, while the limited diversity in V4 is associated with biased positional usage of different donor sites. Using the same or similar numbers of overall donor sites, V2 and V5 are able to generate 2 to 9 times more possible diversity than V1 and V4, which is reflected in direct sequencing measurements. This increase in diversity generation is due to either less positional bias of donor sites or greater proportions of three-segment donor site contributions, or both.

Of note, we measured fewer than 140 full-length TprKs present in any given sample using our filtering criteria, which is substantially less than our theoretical diversity estimates. These measured estimates could be biased by the limited copy numbers (<10,000 copies) available for T. pallidum subsp. *pallidum*-positive clinical specimens and the limited range of copy numbers tested in our study. Conversely, the number of unique variable region sequences and full-length TprKs we identified in each sample could be overestimates due to random sequence errors introduced during PCR amplification or next-generation sequencing. However, we corrected for these sequence errors by requiring each identified variable region sequence to be present in two separate library preparations and using these high-confidence sequences to quality filter the full-length *tprK* sequences.

We also evaluated sequence diversity within the *tprD* locus and its potential to increase TprK diversity within the treponemal population. Whole-genome sequencing of 4 of the Italian strains revealed that the strains contained a limited number of SNVs within the *tprD* locus and no SNVs within any of the 53 identified donor sites. This lack of chromosomal diversity within the 4 strains we analyzed is consistent with the limited genomic diversity present within the global treponemal population (29). Two previously described donor sites (14) each had an SNV compared with the corresponding donor site we identified. This finding indicates that chromosomal mutations can contribute to interstrain TprK diversity; however, when taken with our comparative analysis of the *tprD* locus in 4 of the Italian T. pallidum subsp. *pallidum* strains, mutations within donor sites are unlikely to be a significant contributor to overall TprK diversity in the treponemal population.

Our work was chiefly limited by the few numbers of clinical samples and T. pallidum subsp. *pallidum* strains that have been deeply profiled for TprK diversity. Here, we profiled 13 new T. pallidum subsp. *pallidum*-positive clinical specimens and combined them with 28 previously sequenced samples. However, given the considerable coding potential of TprK, 41 specimens are far too few to understand its overall coding diversity. We also did not explicitly take into account terminal or internal repeats in *tprK* variable regions, which could serve as a mechanism for gene conversion (14). Because of the limited number of total variable regions sampled across these 41 samples (∼10^3^) versus the potential diversity, we considered ourselves underpowered to examine linkages or epistasis between different variable regions. Future work will have to examine whether certain variable region sequences segregate together within a given TprK. The sampling requirements to determine that association are likely quite considerable and beyond the scope of the work presented here.

In addition, we were limited by all 41 strains originating from only 3 geographic locations. The lack of overlap in variable region sequences between the Italian and Chinese strains suggests certain variable region or full-length TprK sequences may be associated with particular geographic regions or sexual networks. Further profiling of *tprK* from a larger collection of strains may reveal associations between multiple geographic regions and sexual networks.

Our work here also does not fully inform how TprK interacts with the immune system. As the overall coding diversity of specific variable regions is somewhat limited, it is possible that epistatic interactions between variable regions could influence epitope structure. Certainly, the paucity of variation across the 41 samples in the V4 region is surprising given that anti-V4 antibodies have been detected in humans (21). We also note that the lower number of measured V3 diversity could be associated with a lack of immunological pressure, especially considering its number of potential donor sites and three-segment gene conversions (21). Alternatively, if there is no or limited epistasis between variable regions and cross-protective antibody is generated against individual variable regions, the diversity-generating potential of individual variable regions combined with the rate of gene conversion could put an upward bound on the time period before T. pallidum subsp. *pallidum* becomes latent in humans.

Our identification of unique variable region sequences and full-length TprK sequences may be impacted by sequence errors introduced during PCR amplification or next-generation sequencing. These sequences errors would result in an overestimation of the number of unique variable region sequences and full-length TprK sequences present in a sample. Our approach of performing short-read sequencing on two separate amplicon preparations for each sample with a high-fidelity polymerase and only including those variable region sequences present in both preparations significantly reduce this possibility. Additionally, we used these high-confidence variable regions from short-read sequencing to quality filter TprK sequences derived from long reads to reduce possible polymerase and sequencing error.

In summary, our work provides a basis for one mechanism of how T. pallidum subsp. *pallidum* maintains lifelong infection, through the constant generation of TprK diversity using a lexicon that approaches that of the baseline human adaptive immune system. Therapeutic interventions that target mechanisms of TprK diversity generation may prove beneficial. We further hypothesize that the loss of the TprK diversity generation will be one of the first changes associated with longitudinal passage of T. pallidum subsp. *pallidum* in the new *in vitro* culture system that provides it respite from constant immune selection.

## MATERIALS AND METHODS

### Sample collection.

Swabs from genital or anal lesions were collected from syphilis patients attending the sexually transmitted infections clinics of Amedeo di Savoia Hospital, University of Turin, and the Ospedale Maggiore in Milan, Italy, from approximately December 2016 to March 2017. The only exclusion criterion for sample collection was an existing record of antibiotic therapy initiated within 30 days from the patient visit. For sample collection, whenever possible, the lesion area was gently squeezed to imbibe the swabs with exudate. The swabs were then placed in sterile microcentrifuge tubes containing 1 ml of 1× lysis buffer (10 mM Tris-HCl, 0.1 M ethylenediaminetetraacetic acid, and 0.5% sodium dodecyl-sulfate) suitable for DNA extraction. The swab shafts were then cut to leave the swab in the buffer. Samples were kept frozen at –80°C until DNA extraction. Sample collection was authorized by the human subject committee of each collecting institution (protocol code PR033REG2016 for the University of Turin; protocol code TREPO2016 for the University of Milan) and informed consent was obtained from each patient. Specimens were then sent as deidentified samples in dry ice to the University of Washington for DNA extraction. Based on the use of deidentified specimens, the University of Washington Institutional Review Board determined this investigation not to be human subject research. Patient demographics were also collected as well as information on sexual orientation, HIV status, syphilis stage, and serology results (VDRL/rapid plasma reagin [RPR] and treponemal hemagglutination [TPHA]/Treponema pallidum particle agglutination assay [TPPA] tests) at the time of patient visit.

### DNA extraction and strain typing.

Frozen samples were thawed at room temperature and vortexed before processing. DNA was extracted from 200 μl of the sample suspension using the QIAamp DNA minikit (Qiagen, Valencia, CA) according to the manufacturer’s instructions. DNA was resuspended in 100 μl of elution buffer provided with the kit. Successful DNA extraction was checked by amplification of a fragment of the human β-globin gene (sense primer, 5′-CAA CTT CAT CCA CGT TCA CC-3′; antisense primer, 5′-GAA GAG CCA AGG ACA GGT A-3′; expected size, 268 bp). Amplifications were performed in a 50-μl final volume using 5 μl of DNA template and 2.5 units of GoTaq polymerase (Promega, Madison, WI). Final concentrations of MgCl_2_, deoxynucleoside triphosphates (dNTPs), and each primer were 1.5 mM, 200 μM, and 0.32 μM, respectively. Cycling conditions were initial denaturation at 95°C for 4 minutes, followed 95°C for 1 min, 60°C for 1 min, and 72°C for 1 min for a total of 40 cycles. Final extension was at 72°C for 5 min.

### Quantification of treponemal load within patient samples.

The treponemal load of each sample was measured by quantitative PCR (qPCR) as previously described (23). Briefly, a portion of *tp47* was amplified using 14.33 μl of 2× QuantiTect multiplex PCR mix, 0.65 μl of 2× QuantiTect multiplex PCR mix with ROX, 0.03 unit of UNG, and the following primers: 5′-CAA GTA CGA GGG GAA CAT CGA T-3′ and 5′-TGA TCG CTG ACA AGC TTA GG-3′. Amplification was monitored with the following probe: 5′-6-carboxyfluorescein (FAM)-CGG AGA CTC TGA TGG ATG CTG CAG TT-nonfluorescent quencher (NFQ)-minor groove minder (MGB)-3′. The following conditions were used for the qPCR: 50°C for 2 minutes, 95°C for 15 minutes, and 45 cycles of 94°C for 1 minute and 60°C for 1 minute.

### Direct from sample amplification and next-generation sequencing of *tprK*.

PCR amplification of *tprK* was conducted using the high-fidelity CloneAmp polymerase (TaKaRa) and *tprK*-specific primers appended to 16-bp PacBio barcodes (see Table S4 in the supplemental material) with 1,000 copies of treponemal DNA input under previously described conditions (23). The resulting 1.7-kb product was cleaned using 0.6× volumes of AMPure XP beads (Beckman-Coulter). For long-read sequencing, library construction and sequencing on a Sequel I single-molecule real-time (SMRT) Cell 1M with a 10-hour movie were completed by the University of Washington PacBio Sequencing Services. A minimum of 5,224 PacBio reads were obtained for each of the samples. Short-read libraries from the same full-length amplicons were constructed with the Nextera XT kit (Illumina), cleaned with 0.6× volumes of AMPure XP beads (Beckman-Coulter), and sequenced on 1 × 192-bp Illumina MiSeq runs. A second replicate of the *tprK* PCR amplification and short-read sequencing was performed as described above to control for potential polymerase error. A minimum of 101,000 Illumina sequencing reads, corresponding to a minimum mean coverage of 6,672×, were obtained for each sample. Sequencing metadata are available in Table S5 in the supplemental material.

10.1128/mBio.02726-20.8 TABLE S4 PacBio-barcoded *tprK* primers used in this study. Download Table S4, XLSX file, 0.01 MB. Copyright © 2020 Addetia et al. 2020 Addetia et al. This content is distributed under the terms of the Creative Commons Attribution 4.0 International license.

10.1128/mBio.02726-20.9 TABLE S5 SRA accessions for sequencing libraries. Download Table S5, XLSX file, 0.01 MB. Copyright © 2020 Addetia et al. 2020 Addetia et al. This content is distributed under the terms of the Creative Commons Attribution 4.0 International license.

### Sequencing analysis of *tprK*.

Analysis of *tprK* was performed using custom python/R scripts available on GitHub (https://github.com/greninger-lab/tprK_diversity). A series of quality-control steps were performed prior to analysis to account for base calling errors during amplification and sequencing of the *tprK* amplicons. For the Italian samples, because of the tagmentation-based library preparation, we quality- (Q20) and adapter-trimmed Illumina reads using Trimmomatic v0.38 (30). Previously published short-read tiling sequencing data for *tprK* from 14 primary and 14 secondary syphilis infections in adults from Xiamen University were downloaded from the NCBI Sequence Read Archive (16, 17). Because of the tiling PCR library design followed by 2 × 300-bp sequencing, both paired-end reads were used in the analysis of the Xiamen samples after adapter trimming using the same options as above. Variable regions were extracted from all samples using fuzzy regular expression matching using 18 bp of neighboring conserved sequence with up to a 3-bp mismatch. For the Italian strains, we included only high-confidence variable region sequences that were present at a read count greater than 5 and a relative frequency greater than 0.1% in both Illumina library preparations in our subsequent analyses. Due to our inability to correct for potential polymerase errors in the Xiamen samples, we required a minimum of 10 reads of support and a relative frequency greater than 0.1% for a given variable region amino acid sequence. We additionally included short-read sequencing data from 2 T. pallidum subsp. pallidum strains passaged in rabbits, which we profiled in a previous investigation (23), in our analysis. Similar to the Italian strains, we performed a technical replicate of *tprK* PCR and short-read sequencing and included only high-confidence variable region sequences that were present at a read count greater than 5 and a relative frequency greater than 0.1%. PacBio Q20 circular consensus sequencing (CCS) reads between 1,400 and 1,800 bp were trimmed of PCR primers using the DADA2 preprocessing pipeline and denoised using RAD (31, 32). Then, we used the high-confidence variable region sequences from the short-read sequencing data to quality check the full-length *tprK* sequences. We required each full-length *tprK* sequence to contain a high-confidence variable region sequence in all 7 of the variable region sequences.

For full-length TprK phylogenetic analysis, we removed any TprK sequences that contained stop codons or which failed to fuzzy match a 20-amino acid region (allowing 3 mismatches) in any conserved region abutting a variable region, which we found was indicative of 2 frame shifts in consecutive variable regions in 2 TprK sequences. The full-length TprK sequences were aligned with MAFFT (33). We next masked the conserved regions of TprK using a fuzzy match which allowed 3 mismatches for regions that were less than 50 amino acids long and 5 mismatches for regions greater than 50 amino acids long. For given sample, any full-length sequences with identical variable region sequences across all 7 variable regions were then merged. A phylogenetic tree was then constructed with FastTree (34) and visualized with the R package ggtree (35).

We used blastn with exact matching over a word size of 10—our estimate of the smallest, high-confidence contribution of a donor site—to identify potential donor sites within a 12.5-kb locus containing the *tprD* gene. We limited the number of potential contributions of each donor site to a variable region to three by restricting the maximum high scoring pairs (-max_hsps 3). We used the subject_besthit option to force nonoverlapping HSPs. In order to generate a list of high-confidence donor sites and reduce putative false positives due to the smaller word size and to control for potential sequencing error, we used only variable regions with greater than 50 reads of support and 0.1% frequency (within-sample) for the Italian strains and greater than 50 reads of support and 0.2% relative frequency for the Xiamen samples and also required donor sites to be used in recovered *tprK* variable region sequences in at least 2 separate samples.

Shannon diversity scores for each sample were calculated using the R package VEGAN (36). Differences in the number of variable region sequences and diversity scores for strains stratified by host factors were assessed using the Wilcoxon rank-sum test.

### Capture sequencing for comparative analysis of the *tprD* locus.

Capture sequencing of 4 specimens, namely, AS9, AS10, AS11, and AS12, was performed as previously described (23). Briefly, pre-enriched libraries were constructed using the KAPA HyperPlus kit (Roche). Hybridization capture using a custom set of biotinylated probes (myBaits; Arbor Bioscience) designed against 3 T. pallidum subsp. *pallidum* reference genomes (GenBank accession no. NC_021508, NC_018722, and NC_016848) was completed to enrich for treponemal DNA. The enriched libraries were purified using 0.8× volumes of AMPure XP beads (Beckman Coulter) and sequenced on a 2 × 300-bp MiSeq run.

A minimum of 1,741,340 sequencing reads were obtained for each sample. Reads were quality and adapter trimmed using Trimmomatic v0.38 (30) and visualized using Geneious v11.1.4 (37).

### Data availability.

Illumina and PacBio reads from *tprK* sequencing of the samples, as well as those from whole-genome sequencing, used in this study are available under the NCBI BioProject number PRJNA589065.
