# Supplementary material for: Estimation of Full-Length TprK Diversity in Treponema pallidum subsp. pallidum
Source: mBio. 2020 Oct 27;11(5):e02726-20. doi: 10.1128/mBio.02726-20 (PMC7593977; doi:10.1128/mBio.02726-20)
Supplement: Data Set S2 [file mbio02726-20_Supp_s2_seq10.html.zip › mbio02726-20_Supp_s2_seq10.html]

Heatmap
