# Supplementary figures and images for: Estimation of Full-Length TprK Diversity in Treponema pallidum subsp. pallidum
Source: mBio. 2020 Oct 27;11(5):e02726-20. doi: 10.1128/mBio.02726-20 (PMC7593977; doi:10.1128/mBio.02726-20)

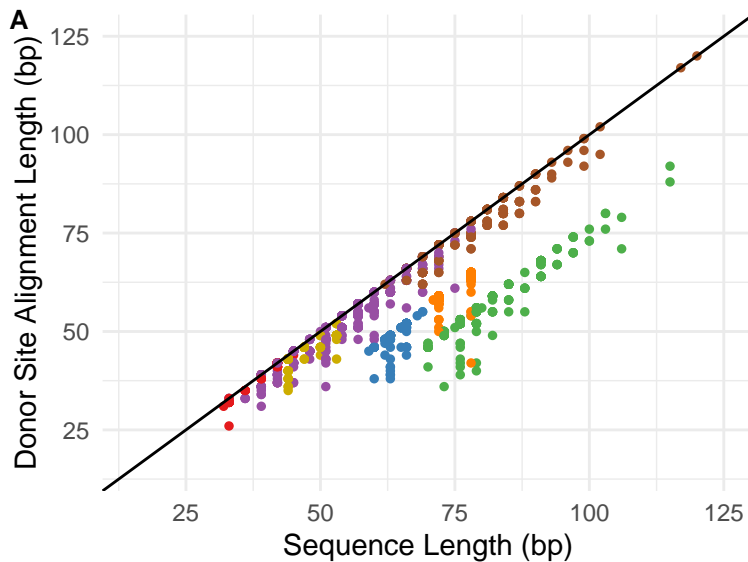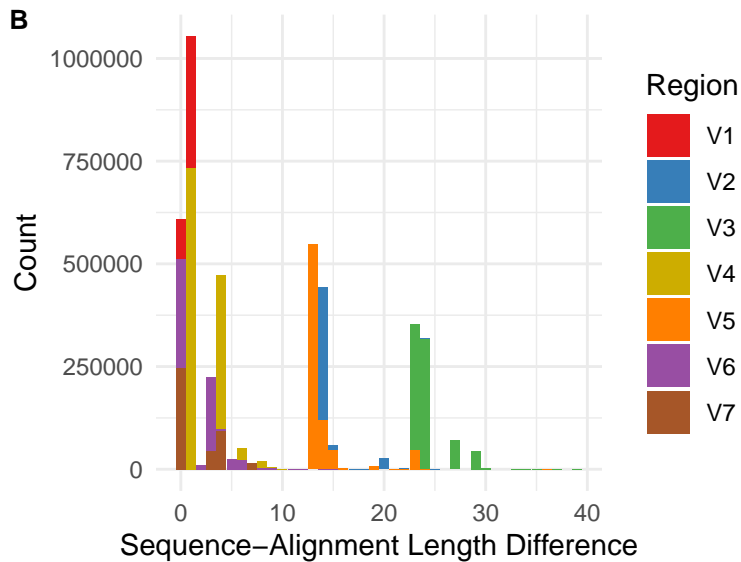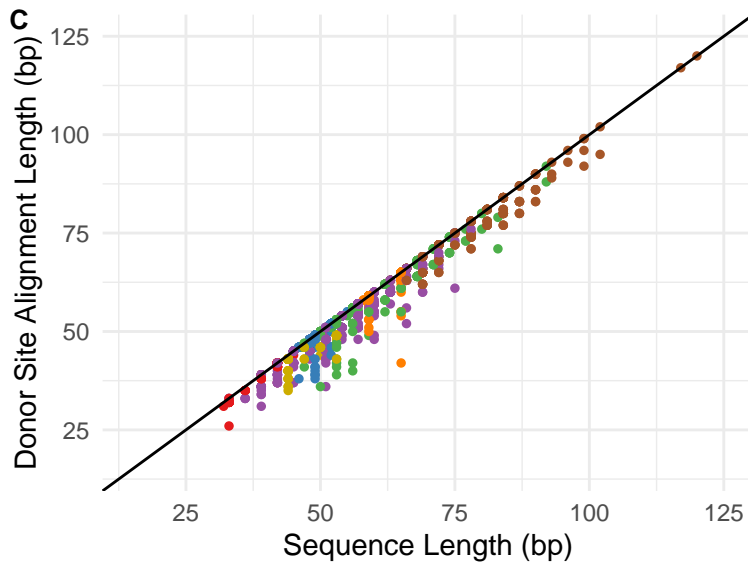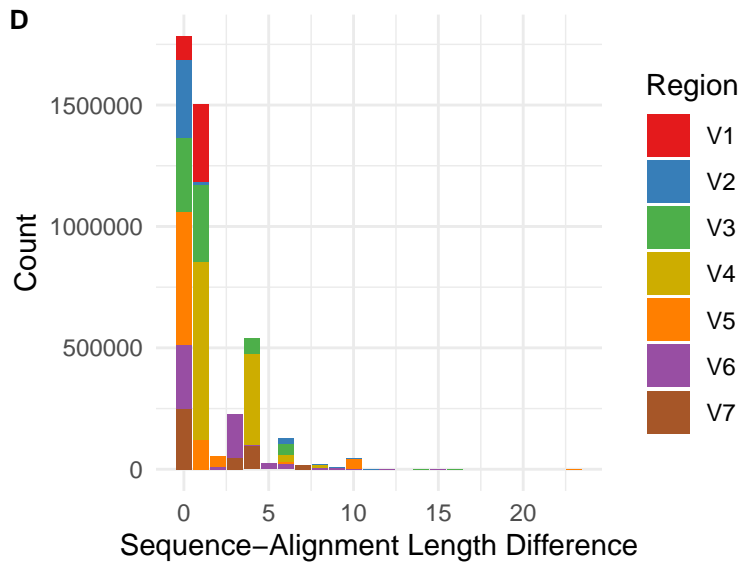

Supplement: FIG S1 [file mBio.02726-20-sf001.pdf]
